# Elucidating sleep disorders: a comprehensive bioinformatics analysis of functional gene sets and hub genes

**DOI:** 10.3389/fimmu.2024.1381765

**Published:** 2024-06-11

**Authors:** Junhan Lin, Changyuan Liu, Ende Hu

**Affiliations:** ^1^ Department of Anesthesiology and Perioperative Medicine, The Second Affiliated Hospital and Yuying Children’s Hospital of Wenzhou Medical University, Key Laboratory of Pediatric Anesthesiology, Ministry of Education, Wenzhou Medical University, Wenzhou, China; ^2^ Laboratory of Anesthesiology of Zhejiang Province, Wenzhou Medical University, Wenzhou, China

**Keywords:** sleep disorders, functional gene sets, hub genes, diagnostic model, drugs

## Abstract

**Background:**

Sleep disorders (SD) are known to have a profound impact on human health and quality of life although their exact pathogenic mechanisms remain poorly understood.

**Methods:**

The study first accessed SD datasets from the GEO and identified DEGs. These DEGs were then subjected to gene set enrichment analysis. Several advanced techniques, including the RF, SVM-RFE, PPI networks, and LASSO methodologies, were utilized to identify hub genes closely associated with SD. Additionally, the ssGSEA approach was employed to analyze immune cell infiltration and functional gene set scores in SD. DEGs were also scrutinized in relation to miRNA, and the DGIdb database was used to explore potential pharmacological treatments for SD. Furthermore, in an SD murine model, the expression levels of these hub genes were confirmed through RT-qPCR and Western Blot analyses.

**Results:**

The findings of the study indicate that DEGs are significantly enriched in functions and pathways related to immune cell activity, stress response, and neural system regulation. The analysis of immunoinfiltration demonstrated a marked elevation in the levels of Activated CD4+ T cells and CD8+ T cells in the SD cohort, accompanied by a notable rise in Central memory CD4 T cells, Central memory CD8 T cells, and Natural killer T cells. Using machine learning algorithms, the study also identified hub genes closely associated with SD, including IPO9, RAP2A, DDX17, MBNL2, PIK3AP1, and ZNF385A. Based on these genes, an SD diagnostic model was constructed and its efficacy validated across multiple datasets. In the SD murine model, the mRNA and protein expressions of these 6 hub genes were found to be consistent with the results of the bioinformatics analysis.

**Conclusion:**

In conclusion, this study identified 6 genes closely linked to SD, which may play pivotal roles in neural system development, the immune microenvironment, and inflammatory responses. Additionally, the key gene-based SD diagnostic model constructed in this study, validated on multiple datasets showed a high degree of reliability and accuracy, predicting its wide potential for clinical applications. However, limited by the range of data sources and sample size, this may affect the generalizability of the results.

## 1 Introduction

Sleep is recognized as an essential physiological requirement for humans, crucial not only for standard physical growth and development but also for the stabilization and integration of memory (1). Sleep disorders (SD) represent a group of conditions characterized by difficulties in initiating sleep, maintaining sleep, or experiencing restorative sleep. The 2017 third edition of the “International Classification of Sleep Disorders” (ICSD-3) by the American Academy of Sleep Medicine classifies SD into seven categories: insomnia, sleep-related breathing disorders, central disorders of hypersomnolence, circadian rhythm sleep-wake disorders, parasomnias, sleep-related movement disorders, and miscellaneous SD (2). In light of the evolving economy and society, factors such as increasing work and life stress, along with lifestyle modifications, have made sleep disorders (SD) a progressively more significant concern. These disorders not only impact an individual’s physical and mental well-being but also have a substantial influence on social and emotional functioning, affecting both adults and children (3). Research into SD is currently in its infancy, and its etiological factors are intricate, encompassing various causative elements such as physiological and psychological aspects, genetic inheritance, body constitution, environmental conditions, social and interpersonal dynamics, mental stimuli, somatic diseases, psychiatric disorders, and adverse drug reactions (4, 5). These factors may induce abnormalities in the brain’s sleep centers and their functions or provoke neurobiochemical alterations, consequently disrupting the structure and process of sleep (6).

A significant proportion of adults persistently fail to meet the recommended sleep duration, despite the growing recognition of the importance of healthy sleep. This renders the enhancement of sleep quality a critical concern for global health policy. Research demonstrates that adults exhibit heightened susceptibility to the impacts of sleep quality and circadian rhythm disruptions, potentially aggravating chronic health conditions (7, 8).

Furthermore, the modern 24/7 lifestyle, coupled with the pervasive use of electronic devices and social media, has precipitated widespread sleep deprivation among children and adolescents, posing potential risks to their neurological development, mental well-being, and cardiovascular health (9). Empirical research also indicates that sleep deprivation is intricately linked with suboptimal cardiac metabolic health, cognitive deterioration, and a heightened risk of dementia in older adults, emerging as a significant modifiable risk factor in contemporary health (10, 11).

In this study, we conducted a comprehensive investigation of physiological functions, expression pathways, and gene expression associated with SD by analyzing datasets from the GEO database. This led to the identification of genes that hold significant diagnostic and therapeutic potential. Based on these findings, we formulated a diagnostic model predicated on hub genes and assessed the efficacy of these genes in discerning SD. The developed model offers substantial references for clinical diagnostics and therapeutics.

## 2 Methods

### 2.1 Data collection and normalization

We retrieved datasets related to SD and their corresponding control groups from the Gene Expression Omnibus (GEO) database (http://www.ncbi.nlm.nih.gov/geo) (12). Human RNA expression data from 17 individuals with SD and 25 healthy controls were obtained from the GSE208668 dataset, derived from the GPL10904 platform. For the validation of the subsequently identified hub genes, we utilized the GSE240851, GSE56931, and GSE98582 datasets, which were sourced from the GPL24676, GPL10379, and GPL6244 platforms, respectively. These datasets included varying numbers of SD patient and control samples. Additionally, the GSE165041 dataset, generated on the GPL18573 platform, comprised microRNA expression data from 10 SD patients and an equal number of healthy controls. All datasets were normalized using the “limma” package within the R software environment, version 4.1.2 (13).

### 2.2 Identification of differentially expressed genes

We utilized the limma package in R software to process the normalized datasets GSE240851 and GSE208668, aiming to identify DEGs. To ascertain statistical significance, DEGs were determined based on |log2fold change| ≥ 0.58 and a false discovery rate <0.05. Subsequently, we employed the ggplot2 package to create volcano plots for the DEGs. Moreover, we selected the top 20 genes, ranked by |log2fold change|, to build heatmaps as part of our analysis.

### 2.3 Gene function enrichment analysis

The “c2.all.v2023.1.Hs.entrez” and “c5.all.v2023.1.Hs.entrez” datasets, which serve as reference gene sets, were downloaded from the GSEA official website originating from the MSigDB database (14). Gene set enrichment analysis (GSEA) was then performed using the “clusterProfiler” package in R software (15). Subsequently, the analysis results were visualized utilizing the “enrichplot” package in R software.

### 2.4 Immune infiltration analysis

We employed the ImmuCellAI and ssGSEA methodologies to estimate the abundance of 24 and 28 distinct types of immune cells, respectively, in the tissues of SD patients and a normal population, thereby enabling precise delineation of immune cell profile differences between the two groups (16, 17). Furthermore, we conducted Spearman correlation analysis to elucidate the interrelationships among the distributions of various immune cells.

### 2.5 ssGSEA

We downloaded the H: hallmark gene sets from the GSEA official website to probe the functional disparities between SD patients and the normal cohort. With these gene sets, we applied the ssGSEA algorithm to evaluate 50 gene sets, aiming to discern potential variances between the two groups. Subsequently, we utilized the Mantel algorithm to analyze the correlations among these gene sets (18). This approach allowed us to investigate the differences in gene expression profiles between the two cohorts and to identify potential molecular pathways associated with the disease.

### 2.6 Protein-protein interaction network construction

For protein interaction analysis, we utilized the STRING 4 online platform and specifically selected PPI pairs with a confidence score greater than 0.40. Subsequently, we employed the Cytoscape V3.9.0 software for visualizing the PPI network (19). Within the network, the significance of each node was determined by calculating their Degree values using the CytoHubba plugin. This analysis allowed us to identify the top 20 pivotal genes based on their ranking of importance (20).

### 2.7 Random forest gene selection

For gene selection, we utilized the Random Forest (RF) algorithm, a binary tree-based recursive partitioning method. The “randomForest” package in R was used, with parameters set to ntree=1000, mtry=3, and importance=true (21). Employing the Gini index as the primary assessment criterion, the Random Forest algorithm was used to rank the DEGs, and the top 20 genes with a significance value greater than 3 were earmarked for further analysis.

### 2.8 Support vector machine gene selection

We utilized the SVM-RFE (Recursive Feature Elimination) approach to optimize the predictive model by minimizing the number of feature vectors produced by the SVM. This approach, being an effective binary classification tool, operates by constructing a classification hyperplane to delineate decision boundaries. In order to enhance the algorithm’s precision, we configured parameters to method=repeatedcv and repeats=10 in the R package “1071”, and employed ten-fold cross-validation. This approach aimed at augmenting the algorithm’s precision (22).

### 2.9 Model construction and evaluation

To develop the LASSO model (23), we integrated genes identified through CytoHubba, RF, and SVM algorithms. This method effectively enabled the identification of critical hub genes for diagnosing SD. We then employed the Logistic regression approach to investigate pivotal factors associated with SD, ultimately constructing a simplified model. Subsequently, we evaluated the classification performance of the model using the Receiver Operating Characteristic (ROC) curve and the corresponding Area Under the Curve (AUC).

### 2.10 Validation of the diagnostic model

In order to evaluate the robustness and general applicability of our developed diagnostic model, we computed the AUC of the ROC curve for the model using three distinct datasets: GSE240851, GSE56931, and GSE98582. This procedure aimed to ascertain the model’s performance across diverse datasets. It was essential to assess its efficacy in different contexts and ensure its capability to perform consistently across varied data sources.

### 2.11 Exploration microRNAs targeting the genes

We utilized the Limma package in R to conduct a differential analysis of the expression matrix from GSE165041, aiming to identify miRNAs that were differentially expressed (DEmiRNAs). Our criteria for significance were miRNAs exhibiting a false discovery rate <0.05 and |log2fold change| >0.5. Moreover, we employed the miRNet database to investigate potential miRNAs associated with differentially expressed genes (DEGs), in order to gain further insight into their involvement in SD (24).

### 2.12 Drug and gene interaction scoring

We obtained data on drugs related to core genes from the Drug-Gene Interaction database (DGIdb) (25). We used the “ggplot2” package in R to create bar charts showing interaction scores, visually indicating the intensity of interactions between various drugs and core genes.

### 2.13 Establishment of animal models

The SD model, following the methodology outlined by Alkadhi and Alhaider (26), was established in 10-month-old male mice. The experimental mice were subjected to SD treatment for a duration of 8 weeks by being placed on a small fixed platform encircled by water, with access only to water and food. Meanwhile, for the control group, another set of mice was housed in a comfortable environment with a 12-hour light/dark cycle and unrestricted access to water and food. All mice received standard pellet feed, with the daily quantity of feed being maintained uniformly across all groups. This experimental design aimed to simulate the effects of SD on physiological functions, thus laying the groundwork for subsequent investigations into gene expression and drug treatment efficacy.

### 2.14 RT-qPCR

The cortex in SD mice was used for total RNA extraction, employing the TransZol Up Plus RNA Kit (TransGEN, Beijing, China) (27). The RNA concentration and quality were then assessed using the Nanodrop Spectrophotometer (Termo Scientifc, Waltham, MA, USA). Subsequently, reverse transcription was carried out using the TransScript^®^ One-Step gDNA Removal and cDNA Synthesis SuperMix (AT311, TransGEN, Beijing, China). Amplification was monitored with the ChamQ Universal SYBR qPCR Master Mix (Novozymes Q711) and a QuantStudio™ 5 Real-Time PCR System (Thermo Fisher Scientific). The internal reference was β-actin, and the relative gene expression was determined using the 2-ΔΔCT formula. Detailed primer sequences can be found in **Table 1**.

**Table 1:** Primer sequences of mRNA for RT-qPCR.

Gene	Primer sequence, 5’–3’ Forward	Reverse
IPO9	CAGTGACAGCCTTGGTGAAA	TCTCCAGTAGGGCATGGACA
RAP2A	CAAACTGTACCACGCCCTCT	GTTGCTAGGTGGATTGGGCT
DDX17	TCTTCAGCCAACAATCCCAATC	GGCTCTATCGGTTTCACTACG
MBNL2	CCCAAAAGTTGCCAGGTTGAA	CTGGGTTTTTAAGTGTGTCGGA
PIK3AP1	CTGGACTCTGCTTCTAACCCC	TGACACCATTCCTCCGCATC
ZNF385A	CAGAACCAAGGGAAGGGGAC	GAAGGGCAGGATCTGCTTGA
β-Actin	GCAGGAGTACGATGAGTCCG	ACGCAGCTCAGTAACAGTCC

### 2.15 Immunoblotting for protein evaluation

Western blotting was performed for IPO9, RAP2A, DDX17, and GAPDH, as described previously (**Table 2**). Enhanced chemiluminescence reagents were used to detect protein expression, and quantitative analysis was conducted using Image J software (28).

**Table 2:** List of the primary antibodies.

Antibody	Catalogue number	Brand	Application	Dilution	Species	MW(kDa)
IPO9	abs134286	absin	WB	1:1000	Rabbit	116
RAP2A	abs105749	absin	WB	1:1000	Rabbit	21
DDX17	abs111924	absin	WB	1:1000	Rabbit	72
GAPDH	GB15002-100	Servicebio	WB	1:2000	Rabbit	36

### 2.16 Statistical analysis

The normality of the data was assessed using the Shapiro–Wilk test. The t-test was utilized to compare the data between two groups, while comparisons among multiple groups were conducted using one-way ANOVA, followed by either the LSD *post hoc* test or Tukey’s *post hoc* test. The statistical analyses were conducted using R software version 4.2.1 and SPSS 25, with significance defined as P < 0.05. Furthermore, all experiments were independently repeated at least three times to ensure the validity of the results.

## 3 Results

### 3.1 Identification of DEGs

**Figure 1:**
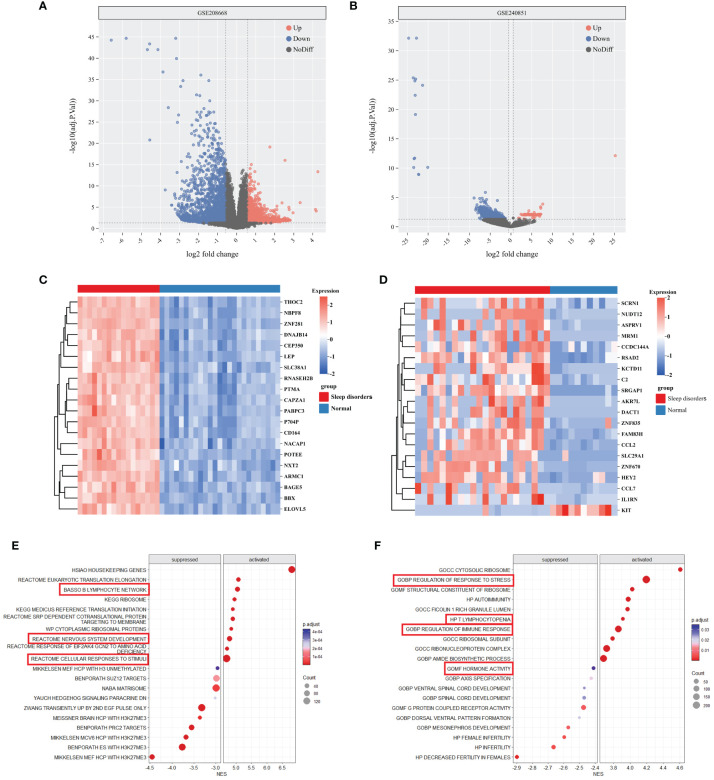
DEGs and enrichment analysis of SD. **(A, B)** Volcano plot of DEGs between Sleep disorders and Normal groups in GSE208668 and GSE240856. **(C, D)** Heatmap of top 20 DEGs in GSE208668 **(C)** and GSE240856 **(D)**. **(E, F)** The top 10 gene sets that areactivated or inhibited in the C2 **(E)** and C5 **(F)** gene sets of MSigDB.

In the GSE208668 and GSE240851 datasets, a differential expression analysis was conducted on SD samples and normal control samples. The analysis identified 5964 DEGs in the GSE208668 dataset and 1375 DEGs in the GSE240851 dataset. Utilizing these DEGs, volcano plots (**Figures 1A, B**) were constructed, and the top 20 genes with the highest |log2fold change| values were selected for heatmap generation (**Figures 1C, D**). These results demonstrate the effectiveness of DEGs in distinguishing between the SD group and the normal control group.

### 3.2 Functional enrichment analysis of DEGs

The differential gene analysis of GSE208668, using MSigDB’s C2 and C5 gene sets, unveiled substantial differential expression in gene clusters relevant to immune cell functions, stress responses, and nervous system activities. Specifically, within the C2 gene set, activation of the gene cluster associated with nervous system development was observed, while the gene cluster linked to the B lymphocyte network was suppressed. In the C5 gene set, gene clusters involved in stress response regulation exhibited a downregulated trend, indicating a potential imbalance in environmental stress response regulation. Additionally, gene clusters associated with spinal cord development demonstrated differential expression. Moreover, immune-related gene clusters, including those implicated in autoimmunity and T-cell deficiency, exhibited alterations in expression levels (**Figures 1E, F**). Further comprehensive details can be found in **Supplementary 1**.

The results of the GSEA analysis demonstrated significant enrichment of terms in KEGG pathways closely associated with neurodegenerative diseases and metabolic pathways, such as Alzheimer’s disease, leishmaniasis infection, lysosomal function, oxidative phosphorylation, Parkinson’s disease, and viral myocarditis. Notably, major signaling factors involved in immune regulation, including IL-4, IL-8, and IL-12, were also found to be significantly enriched in various immune system pathways. Furthermore, signaling pathways related to neural signaling, including neurotrophic factor signaling, B cell receptor signaling, and tumor necrosis factor α signaling, demonstrated significant enrichment **Supplementary 2**.

### 3.3 Analysis of immune cell infiltration

**Figure 2:**
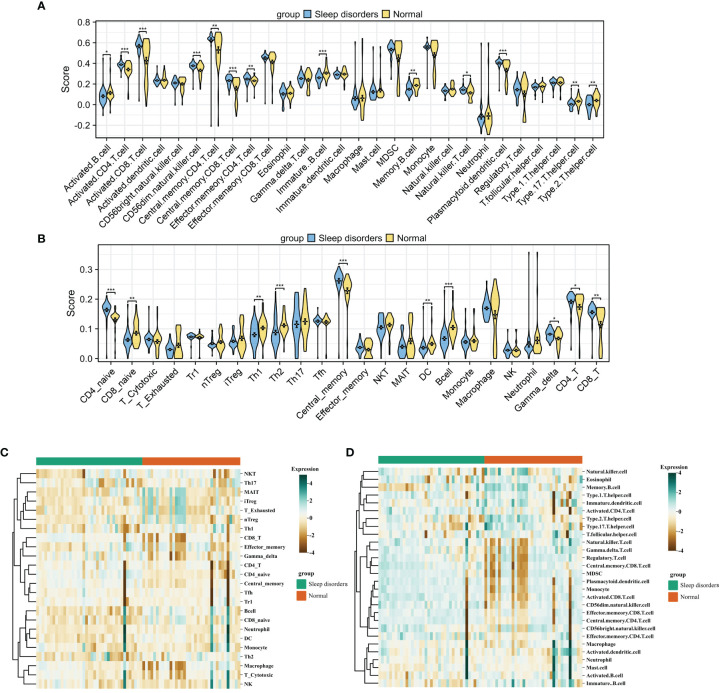
Immune Cell Infiltration **(A, B)** Violin plot comparing the results of two immune infiltration algorithms. ssGSEA **(A)** and ImmuCellAI **(B)**. **(C, D)** Heatmap of the proportions of two immune infiltration algorithms in the Sleep disorders and Normal groups. ImmuCellAI **(C)** and ssGSEA **(D)**. * p < 0.05, ** p < 0.01, *** p < 0.001.

**Figure 3:**
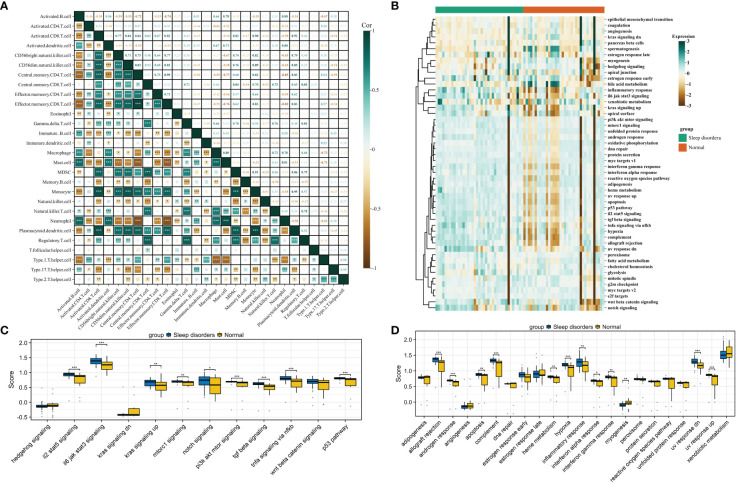
Gene set scoring. **(A)** Correlation graph for 28 types of immune cells. **(B)** Heatmap of scores for 50 gene sets. **(C)** Box plot comparing scores of 12 signaling pathways between Sleep disorders and Normal groups. **(D)** Box plot comparing scores of 22 physiological functions between Sleep disorders and Normal groups. * p < 0.05, ** p < 0.01, *** p < 0.001.

After integrating the GSE208668 and GSE240851 datasets and mitigating batch effects, we observed significant discrepancies in the distribution of various immune cell types between patients with systemic lupus erythematosus (SD) and the normal control group. This was confirmed by employing two distinct immune infiltration scoring methods (**Figures 2A–D**). Notably, the infiltration scores for Activated CD4+ T cells and Activated CD8+ T cells were substantially higher in patients with SD compared to the normal group. Moreover, central memory CD4 T cells, central memory CD8 T cells, and natural killer T cells exhibited an increasing trend in SD patients. In contrast, the infiltration scores of Activated B cells, Immature B cells, Th17 cells, and Th2 cells demonstrated a declining trend in patients with SD relative to the normal group. Correlation analysis based on both algorithms indicated a level of concordance. Specifically, CD8+ T cells and memory T cells consistently showed a strong positive correlation, while neutrophils and regulatory T cells exhibited a negative or non-significant correlation (**Figure 3A**). For a detailed correlation analysis of the ImmuCellAI algorithm scores, please refer to **Supplementary 3**.

### 3.4 Gene set scoring based on ssGSEA

The heatmap in **Figure 3B** displays the scores for 50 gene sets, from which we chose 12 related to signaling pathways and 22 associated with physiological functions for further investigation. A comparative analysis of the gene set scores between the groups revealed that in the SD group, median scores for gene sets such as androgen response, apoptosis, complement, hypoxia, and inflammatory response were significantly higher, while the median score for the myogenesis gene set was notably lower compared to the normal group. Additionally, significant disparities were observed in signaling pathways such as il2 stat5 signaling, il6 jak stat3 signaling, the p53 pathway, and tgf beta signaling (**Figures 3C, D**).

We then focused on 12 gene sets with significant differences in physiological functions between the groups for further correlation analysis. These analyses revealed that, except for the inflammatory response gene set, other gene sets showed significant correlations with immune infiltration and signaling pathway scores. Moreover, the scores within these 12 biological function gene sets also exhibited a high degree of correlation (**Supplementary 4**). For detailed results on pathway scoring and physiological function scoring correlations, please refer to **Supplementary 5**.

### 3.5 Identification of key genes in PPI network

**Figure 4:**
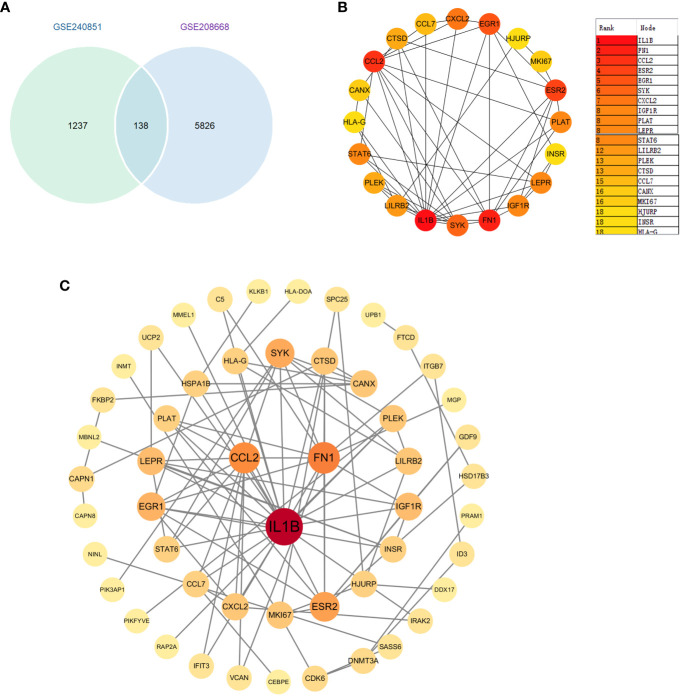
**(A)** Venn diagram of differential genes between datasets GSE20851 and GSE208668. **(B)** Identification of genes in the PPI network common to differential genes. **(C)**.Top 20 genes selected by the CytoHubba plugin.

We first identified 138 co-expressed differential genes by intersecting the DEGs from the GSE208668 and GSE240851 datasets (**Figure 4A**). Subsequently, 52 genes were selected for network visualization in Cytoscape using the STRING database for PPI analysis with a medium confidence threshold of 0.4 (**Figure 4B**). To identify the top 20 key genes within the PPI network, the CytoHubba plugin in Cytoscape was employed (**Figure 4C**).

### 3.6 Utilizing machine learning for enhanced feature gene selection

**Figure 5:**
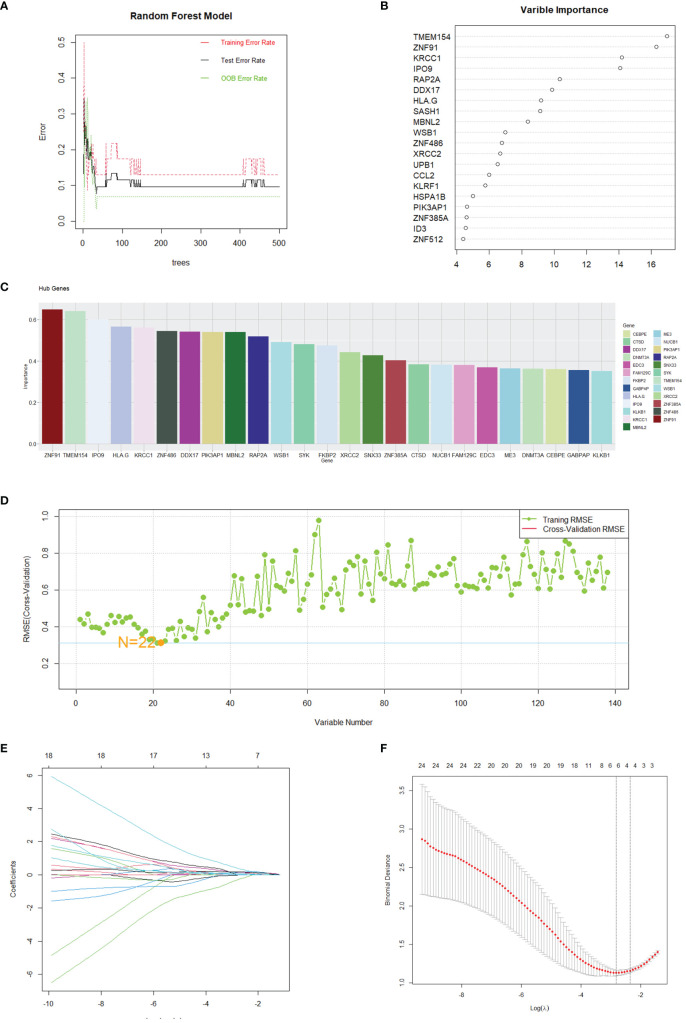
Gene selection through machine learning. **(A)** The correlation plot between the number of Random Forest trees and model error. **(B)** Top 20 genes selected by the RF method. **(C)** Top 25 genes identified by SVM, ranked by the percentage decrease in mean impurity. **(D)** Results obtained from the predictive model of the Root Mean Square Error through cross-validation. **(E, F)** Cvfit and lambda curves demonstrating the use of the LASSO regression, performed with the minimum criteria.

The assessment of hub genes started with the use of the GSE208668 dataset and the RF algorithm, which led to the identification of 138 co-expressed differential genes. The model’s performance was then evaluated across a spectrum of tree quantities, showing a gradual decrease in the error rate as the number of trees increased, ultimately indicating enhanced stability and reliability (**Figure 5A**). Following this, the feature importance was assessed through the calculation of the percentage decrease in mean impurity (%IncMSE), which resulted in the identification of the top 20 genes that had the most significant contributions to the model’s prediction (**Figure 5B**). Subsequently, the SVM algorithm was utilized to determine the hierarchical importance of the 138 genes (**Figure 5C**). The model’s predictive efficacy was then measured using the Root Mean Square Error (RMSE) through cross-validation methodologies, as shown in **Figure 5D**. The results indicated that the model demonstrated its strongest predictive capability when N was set to 22, leading to the selection of these 22 genes for further analysis.

### 3.7 Development and verification of the diagnostic model

After identifying 40 key genes through the amalgamation of genes identified via CytoHubba, Random Forest, and SVM (**Supplementary 6** for details), LASSO regression was utilized to select diagnostic genes. This process involved adjusting the regularization parameter λ and observing its effects on coefficient estimation (**Figure 5E**). The optimal λ value was determined through the ten-fold cross-validation method (**Figure 5F**). In the final analysis, diagnostic model construction involved the selection of IPO9, RAP2A, DDX17, MBNL2, PIK3AP1, and ZNF385A.

**Figure 6:**
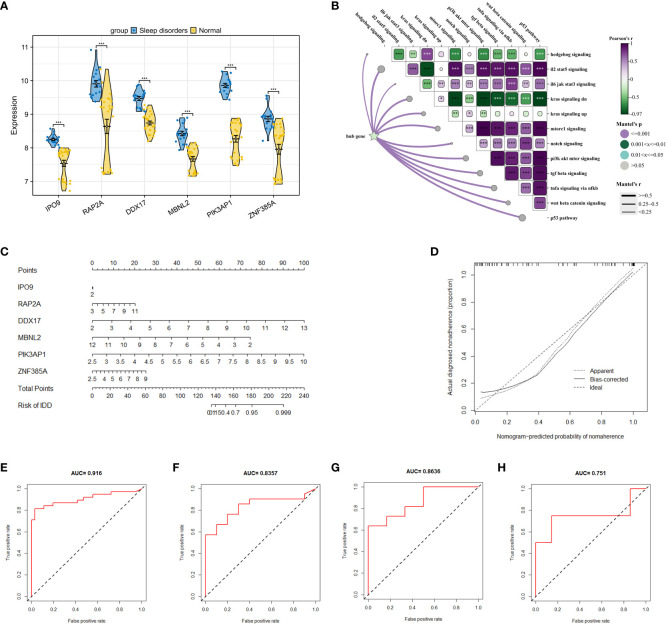
Construction of a research and diagnostic model based on hub genes. **(A)** Expression of 6 hub genes in dataset GSE208668. **(B)** Correlation between 6 key genes and crucial signaling pathways. **(C)** A nomogram model, incorporating 6 hub genes, was constructed to predict risk. **(D)** The calibration curve of the nomogram to test the predictive performance of the model. **(E)** ROC curves analysis of GSE208668 for the diagnostic model. **(F–H)** display ROC curve analyses of the diagnostic model applied to GSE240851, GSE98582, and GSE56931 datasets. * p < 0.05, ** p < 0.01, *** p < 0.001.

When compared to the normal group, these 6 hub genes demonstrated significant disparities (**Figure 6A**) and significant correlations with scores of biological function genes (**Figure 6B**). The interactions between hub genes and other functional scores are presented in **Supplementary 7**. A nomogram was constructed based on these 6 genes, with each gene correlating with a specific scoring criterion (**Figure 6C**). The calibration curve of the nomogram indicated commendable predictive performance of the model (**Figure 6D**). ROC curve analysis further demonstrated the substantial diagnostic value of these genes with an overall AUC value of 0.916 (**Figure 6E**). To corroborate the accuracy of the model, three independent datasets were utilized in logistic regression analysis. The AUC values for GSE240851, GSE98582, and GSE56931 were 0.835, 0.863, and 0.751, respectively, demonstrating the stability and reliability of the model in diagnosing SD (**Figures 6F–H**).

### 3.8 Investigating miRNAs and prospective therapeutic agents

**Figure 7:**
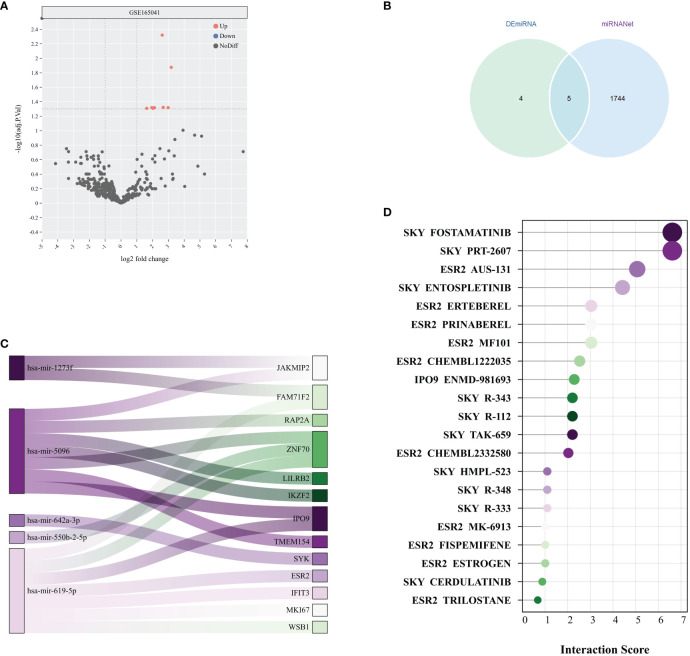
Potential therapeutic drug search based on miRNA. **(A)** Volcano plot of miRNA differential analysis results from the GSE165041 dataset. **(B)** Venn diagram of DEmiRNAs and miRNAs obtained from miRNANet. **(C)** The Sankey plot shows the relationships between 5 miRNAs and their target genes. **(D)** Interaction scores between genes and drugs were obtained from the DGIdb database.

The analysis of the GSE165041 dataset revealed 9 upregulated DEmiRNAs that distinguished the SD group from the normal group, as evidenced in **Figure 7A**. Subsequently, an exploration of the association between these DEmiRNAs and the previously identified 138 DEGs was conducted using the miRnet database, resulting in the identification of 5 miRNAs with potential as therapeutic targets, as depicted in **Figure 7B**. Following this, a validation of the potential therapeutic efficacy of the 5 identified miRNAs and their corresponding key mRNAs was performed, as shown in **Figure 7C**. Subsequently, compounds with high interaction scores with the key genes were identified utilizing the DGIdb, as illustrated in **Figure 7D**. These findings indicate that the identified miRNAs and compounds might serve as potential therapeutic agents for the treatment of SD. Nevertheless, it is essential to note that further research is necessary to substantiate their efficacy and safety.

### 3.9 Analysis of predicted gene expression in brain tissue of SD mice

**Figure 8:**
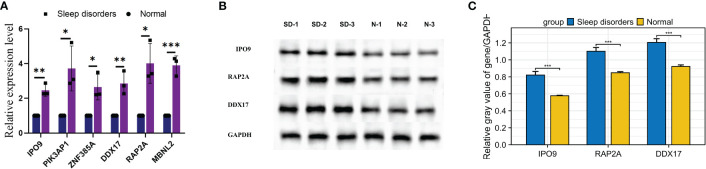
Expression of mRNA and proteins in mice with SD. **(A)** Relative mRNA expression of the 6 hub genes. **(B)** Western blot results for 3 relative proteins. **(C)** Relative protein expression. * p < 0.05, ** p < 0.01, *** p < 0.001.

RT-qPCR was used to scrutinize the expression levels of 6 hub genes in the tissue. The mRNA expression levels of 6 genes in the SD group showed a significant elevation relative to the normal group, as illustrated in **Figure 8A**. Moreover, Western blotting analysis revealed significant elevations in the protein levels of genes such as IPO9, RAP2A, and DDX17 within the cortical regions of mice suffering from SD, as depicted in **Figures 8B, C**. These findings align with previous bioinformatics analysis outcomes, providing further support for the correlation of expression levels among these 6 hub genes.

## 4 Discussion

Epidemiological research has revealed that SD are prevalent worldwide, with industrialized countries exhibiting a particularly high prevalence (29). The correlation between SD and a range of conditions, including cardiovascular diseases, diabetes, and mental disorders, has been extensively documented. This emphasizes the critical role of sleep quality in maintaining overall health. The integration of genomics, transcriptomics, and proteomics has significantly advanced the understanding of the molecular mechanisms underlying SD, leading to the discovery of numerous genes and molecular pathways associated with these disorders (30, 31). Particularly in terms of genetics, studies by Lee YY et al. have confirmed that genes play an important role in the development of SD (32). The etiology of SD involves a complex interplay of biological, psychological, and social factors, with principal neurobiological mechanisms comprising substances and regulators, genetic factors, lifestyle choices, and light exposure (33, 34).

This research used advanced bioinformatics approaches to analyze differentially expressed genes (DEGs) between patients with SD and a normal group, revealing significant differences in immune response, stress response, and nervous system development. Notably, the study uncovered a close correlation between inflammatory pathways (such as IL-2, IL-8, IL-12) and pathways relevant to neurodegenerative diseases. The intimate association between sleep and the immune system suggests that SD may result in immune dysregulation (35). Murine models have shown that SD can influence the signaling of GM-CSF (Granulocyte-Macrophage Colony-Stimulating Factor) by regulating Th17 and activated CD4 T cells (36), which in turn interacts with myeloid cells, exacerbating the progression of autoimmune diseases. Simple sleep deprivation in rats has been found to increase Natural Killer (NK+) and T cells (CD8+) in the spleen and decrease B cells (37). Moreover, research indicates that patients with central hypersomnia exhibit significantly higher levels of activated CD4+ and CD8+ T cells in both peripheral blood and cerebrospinal fluid compared to healthy controls (38). These findings align with previous studies and reveal substantial alterations in activated CD4+ T cells, CD8+ T cells, central memory CD4 and CD8 T cells, natural killer T cells, activated B cells, immature B cells, Th17 cells, and Th2 cells in patients with SD. These results suggest that T cell-mediated autoimmune responses may contribute to the pathogenesis of SD, and the increase in memory T cells and other immune cells implies their intricate involvement in SD. All of these studies corroborate with the results of our analysis, suggesting the existence of complex physiological mechanisms of immune cells in SD. Furthermore, the alterations in B-cell and Th-cell infiltration scores identified in our research provide a new perspective for comprehensive investigation into the roles of these cells in the context of SD.

The implementation of gene set scoring methodologies has elucidated the expression patterns of distinct gene sets in various diseases, thereby aiding in the comprehension of the underlying biological processes and pathological mechanisms (39). A multitude of studies have demonstrated a close association between SD and diverse biological and pathological processes (40). For instance, research conducted by Séverine Lamon et al. found that total SD results in reduced testosterone levels and muscle protein synthesis in patients’ plasma, while the mouse experiments by Yin Cao et al. observed excessive autophagy and apoptosis in hippocampal neuronal cells, concomitant with the activation of the PI3K/AKT signaling pathway (41, 42). Similarly, the study by Yongmei Li et al. indicates that SD leads to the upregulation of autophagy-related proteins, and Anna Brzecka et al. highlight that intermittent hypoxia resulting from SD may elevate the risk of certain cancers (43, 44). The research by Zhong Wang et al. demonstrates that SD may precipitate gut microbiota imbalance and cognitive function decline, with observations of the activation of the Toll-like receptor 4/nuclear factor-κB signaling pathway in mice with transplanted SD microbiota (45). In the context of signal pathway scoring, sleep deprivation has been found to activate the P53 pathway. This activation induces the expression of apoptotic proteins, such as Bax and Bcl-2, leading to the onset of tongue cancer (46). In a molecular mechanism study exploring the link between sleep and breast cancer, a positive correlation was identified between TGF-β and CRP levels and insomnia, while IL-6 showed a negative correlation with sleep-inducing medications (47). Such gene sets have substantiated the results of our bioinformatics research.

Our study findings uncovered a substantial correlation between the scores of 12 signaling pathways and various biological gene sets. Specifically, our results emphasized the significant association of the hedgehog and kras signaling pathways with various biological processes, suggesting their potential contribution to the onset of SD. Additionally, the il2, il6, notch, and PI3K/AKT/mTOR pathways also demonstrated relevance, signifying their potential as crucial areas for further investigation in SD studies. The above findings, which have been mentioned in other studies as having a potential link to SD (48).

Leveraging a comprehensive analysis of differential genes in the GEO dataset and various machine learning algorithms, our study identified 6 pivotal genes - IPO9, RAP2A, DDX17, MBNL2, PIK3AP1, and ZNF385A. These genes are instrumental in the identification of biomarkers and potential therapeutic targets for SD.

The IPO9 gene plays a key role in mediating the docking process of the importin/substrate complex with the nuclear pore complex, enabling the transport of the complex through the pore by binding to nucleoporin proteins using energy-dependent and Ran-dependent mechanisms (49). Research has indicated that the expression of IPO9 is regulated by m6A modification sites, which may be closely linked to the pathogenesis of obesity (50). RAP2A, a member of the RAS oncogene family, encodes a crucial protein that is essential for the activation of cAMP-dependent PKA and ERK signaling pathways. Additionally, it is involved in a signaling complex consisting of NEDD4, RAP2A, and TNIK, which regulates the growth and differentiation of neuronal dendrites (51). Furthermore, RAP2A is implicated in multiple signaling cascades, including cytoskeletal rearrangement, cell migration, adhesion, and proliferation. It also exhibits abnormal expression in various tumors, such as breast, liver, and gastric cancers (52). The DDX17 gene encodes a DEAD box protein and is involved in multiple cellular processes that require alterations in RNA secondary structure (53). Research by Samaan et al. has demonstrated that DDX17 plays a significant role in estrogen and testosterone signaling pathways, influencing the use of alternative promoters in estrogen-responsive genes and affecting the transcription and splicing of many steroid hormone target genes (54). Changes in DDX17 expression may affect mRNA processing of hormones and neurotransmitters associated with sleep regulation, thereby modulating sleep cycle and quality. This finding not only explains the physiological role of DDX17, but may also guide future personalized treatment strategies for SD patients. MBNL2, a member of the muscleblind protein family, encodes a C3H type zinc finger protein that regulates the selective splicing of pre-mRNA (55). Knockout of the MBNL2 gene in mouse models has been associated with diabetes-related characteristics in the central nervous system, including abnormal rapid eye movement sleep tendencies and spatial memory deficits. Furthermore, these mice exhibited delayed recovery and prolonged sleep duration following general anesthesia when compared to wild-type mice (56). PIK3AP1 plays a crucial role in diverse inflammatory responses and the regulation of signal transduction. It connects B cell receptor signaling with the PI3K-Akt signaling pathway, facilitating signal transduction relevant to B cell development. It also links toll-like receptor signaling with PI3K activation, hence helping to prevent excessive production of inflammatory cytokines (57). ZNF385A, a zinc finger protein, modulates the activity of p53/TP53 through direct protein interactions, leading to cell cycle arrest. Emerging studies suggest that this gene may be associated with the decline in cognitive function in the elderly (58, 59). Finally, through the analysis of mRNA and its corresponding protein expression in a mouse model of SD, the involvement of these 6 genes in sleep disturbances was confirmed, revealing significant differences in their expression under diseased conditions.

In our study, using the miRNAnet platform, we identified 5 critical miRNAs targeting 13 genes implicated in SD. Among these findings, hsa-miR-5096, mediated by exosomes, stood out due to its potential to augment the heterogeneity of somatostatin receptors (60). Additionally, a strong correlation was observed between miR-642a-3p and the metabolic levels of adenosine and creatine in the metabolic analysis of varicose veins (61). Furthermore, our research integrated the DGIdb analysis, revealing the potential therapeutic value of drugs such as MF101, ENTOSPLETINIB, FISPEMIFENE, and FOSTAMATINIB. For instance, MF101 has the ability to selectively modulate estrogen receptor β, possibly aiding in the improvement of vasomotor symptoms and enhancing sleep quality in menopausal women (62). Similarly, FISPEMIFENE, a tissue-specific estrogen agonist/antagonist, is also considered beneficial for improving sleep quality, as evidenced in previous research (63).

Our study has yielded potential insights into the molecular mechanisms of SD; however, it is important to acknowledge its limitations. Firstly, our analysis relies on secondary data from existing databases, which raises potential concerns about data quality and representativeness. Furthermore, despite the identification of numerous genes and pathways associated with SD, there is a need for further investigation into the causal relationships and specific mechanisms of action among them. Lastly, our study primarily focuses on the genetic level and does not comprehensively account for the potential impacts of proteins, metabolites, and other non-coding RNAs. Future research efforts should concentrate on validating the biological and clinical significance of these findings, as well as further delineating the specific roles of non-genetic factors in the pathogenesis of SD.

Our research leverages cutting-edge bioinformatics tools to elucidate the genetic underpinnings of sleep disorders, representing a significant advantage in terms of technological application and data handling capacity. The multi-dataset validation process serves as a robust proof of concept, indicating that our findings have a high potential for generalizability across different populations. However, the study is not without its limitations. The dependence on publicly available genomic databases might limit our insights to the data quality and completeness of these resources. Furthermore, while our model shows promising results in computational validations, actual clinical utility will need to be established through prospective clinical trials involving diverse patient demographics to address the varying manifestations of sleep disorders.

Unlike previous studies on single genes and sleep disorders, this study combined bioinformatics to screen key genes and construct a diagnostic model for sleep disorders based on machine learning and big data modeling, which was finally validated on an animal model. Our investigation delves into the pathogenesis of SD from multifaceted perspectives, including genetics, physiology, and pharmacology. Through our analysis, we have discerned that specific gene expression patterns under diverse physiological and pathological conditions may either mitigate or exacerbate disease progression. This influence occurs through their impact on immune responses, epigenetic regulation, and numerous synergistic regulatory mechanisms. The interactions between these factors significantly contribute to our understanding of the complex nature of SD.

## Data availability statement

The datasets presented in this study can be found in online repositories. The names of the repository/repositories and accession number(s) can be found in the article/**Supplementary Material**.

## Ethics statement

Ethical approval was not required for the study involving humans in accordance with the local legislation and institutional requirements. Written informed consent to participate in this study was not required from the participants or the participants’ legal guardians/next of kin in accordance with the national legislation and the institutional requirements. The animal study was approved by laboratory animal ethics committee of Wenzhou Medical University. The study was conducted in accordance with the local legislation and institutional requirements.

## Author contributions

JL: Data curation, Software, Validation, Visualization, Writing – original draft, Writing – review & editing, Conceptualization, Investigation, Methodology, Project administration, Supervision. CL: Data curation, Formal analysis, Software, Supervision, Validation, Writing – review & editing. EH: Conceptualization, Investigation, Project administration, Resources, Supervision, Visualization, Writing – original draft, Writing – review & editing.
