# Supplementary figures and images for: Elucidating sleep disorders: a comprehensive bioinformatics analysis of functional gene sets and hub genes
Source: Front Immunol. 2024 Jun 11;15:1381765. doi: 10.3389/fimmu.2024.1381765 (PMC11196417; doi:10.3389/fimmu.2024.1381765)

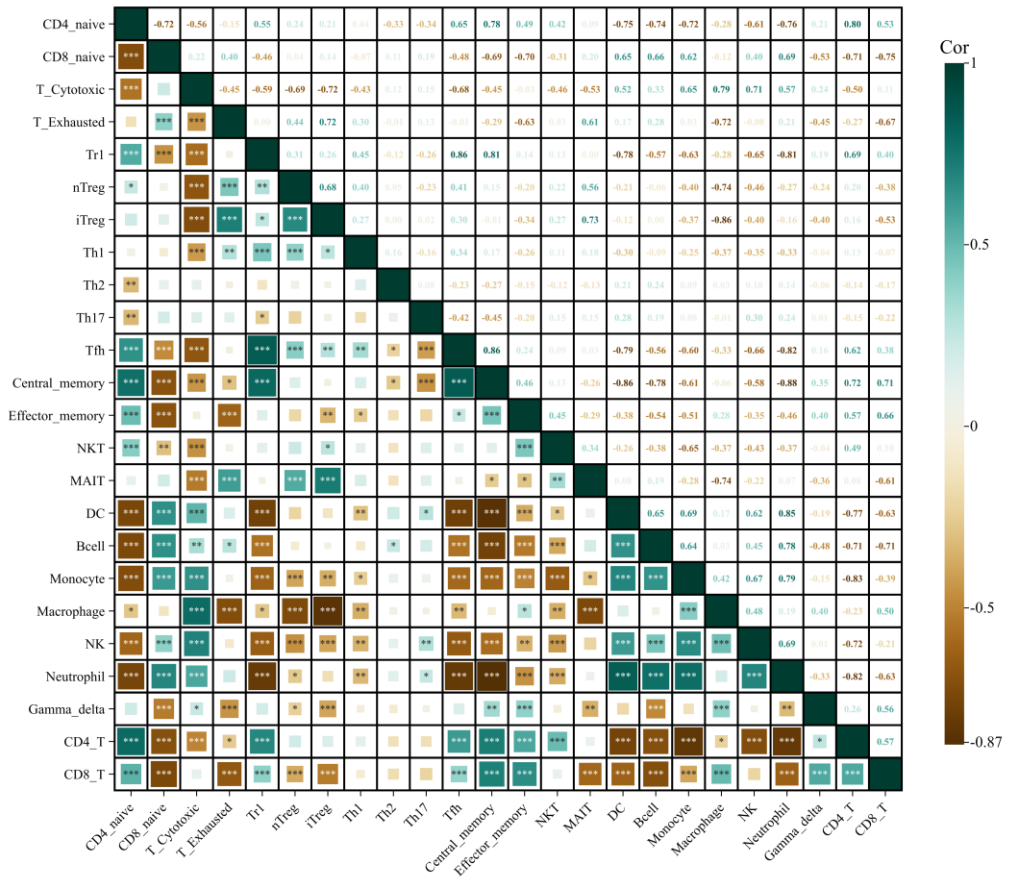

Detailed correlation analysis of the ImmuCellAI algorithm scores, \* $p < 0.05$ , \*\* $p < 0.01$ , \*\*\* $p < 0.001$

Supplement: Supplementary file 1 [file DataSheet_1.pdf]

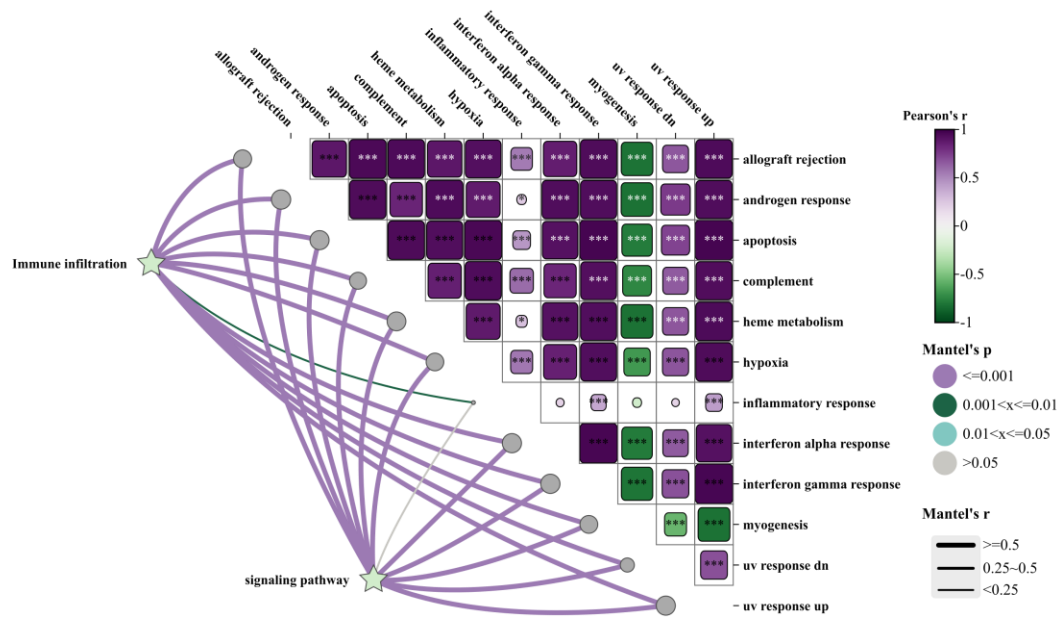

Correlation among immune infiltration, signaling pathways, and key functional gene sets. \* $p < 0.05$ , \*\* $p < 0.01$ , \*\*\* $p < 0.001$

Supplement: Supplementary file 2 [file DataSheet_2.pdf]

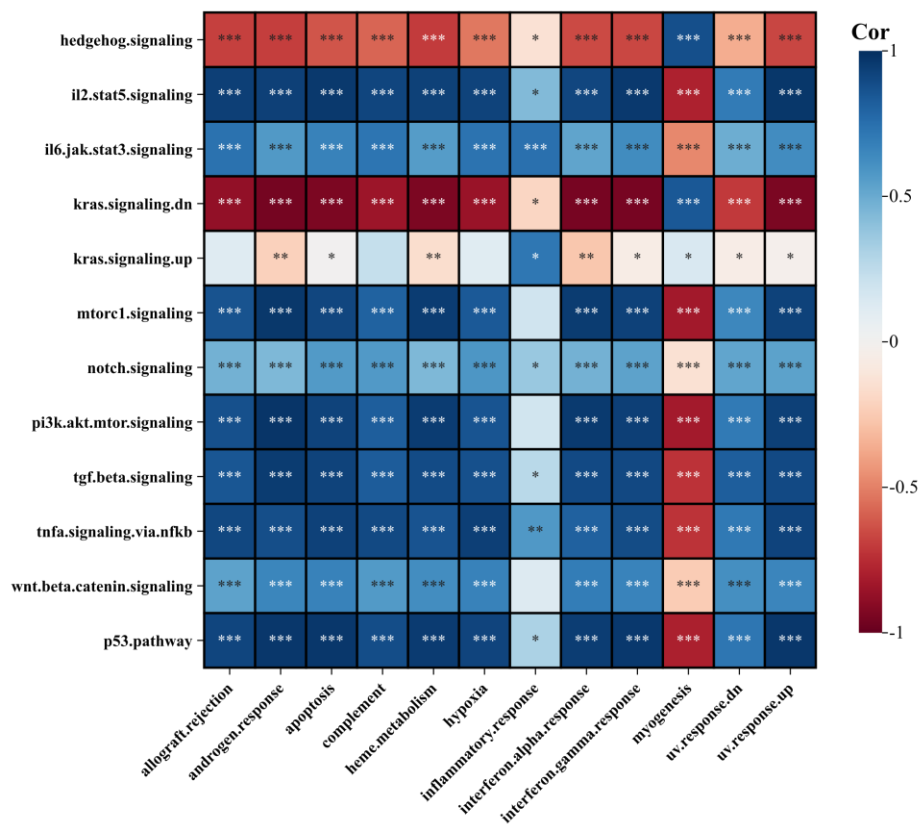

Pathway scoring and physiological function scoring correlations. \*p < 0.05, \*\*p < 0.01, \*\*\*p < 0.001

Supplement: Supplementary file 3 [file DataSheet_3.pdf]

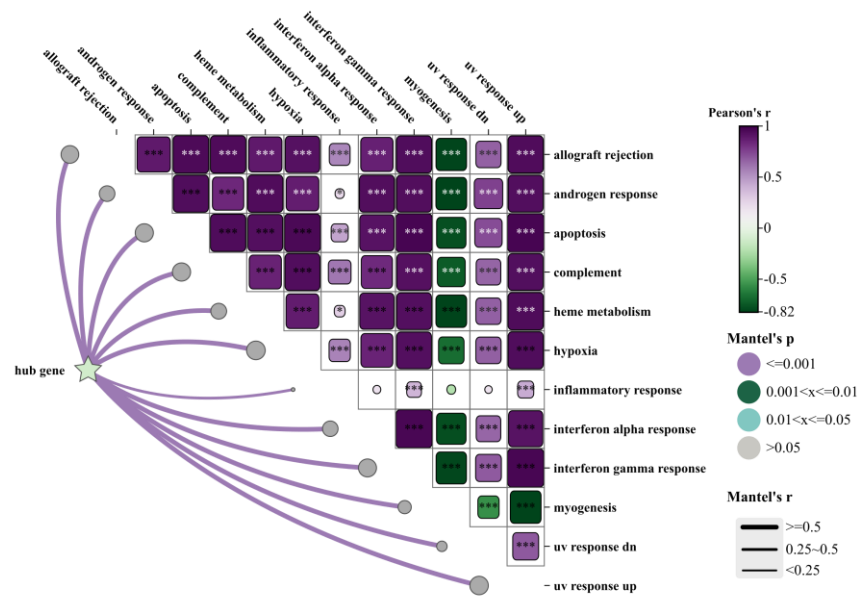

Correlation between hub genes and key physiological functions. \* $p < 0.05$ , \*\* $p < 0.01$ , \*\*\* $p < 0.001$

Supplement: Supplementary file 4 [file DataSheet_4.pdf]
